# Radiation Boost After Adjuvant Whole Breast Radiotherapy: Does Evidence Support Practice for Close Margin and Altered Fractionation?

**DOI:** 10.3389/fonc.2020.00772

**Published:** 2020-06-26

**Authors:** Stephanie Gulstene, Hamid Raziee

**Affiliations:** ^1^Department of Radiation Oncology, University of Western Ontario, London, ON, Canada; ^2^Department of Radiation Oncology, BC Cancer Surrey, University of British Columbia, Vancouver, BC, Canada

**Keywords:** boost radiation, hypofractionated, close margins, radiation therapy (radiotherapy), breast cancer, breast malignancy, breast carcinoma (BC)

## Abstract

Adding a boost to whole breast radiation (WBI) following breast-conserving surgery (BCS) may help improve local control, but it increases the total cost of treatment and may worsen cosmetic outcomes. Therefore, it is reserved for patients whose potential benefit outweighs the risks; however, current evidence is insufficient to support comprehensive and consistent guidance on how to identify these patients, leading to a potential for significant variations in practice. The use of a boost in the setting of close margins and hypofractionated radiotherapy represents two important areas where consensus guidelines, patterns of practice, and current evidence do not seem to converge. Close margins were previously routinely re-excised, but this is no longer felt to be necessary. Because of this recent practice change, good long-term data on the local recurrence risk of close margins with or without a boost is lacking. As for hypofractionation, although there is guidance recommending that the decision to add a boost be independent from the whole-breast fractionation schedule, it appears that patterns-of-practice data may show underutilization of a boost when hypofractionation is used. The use of a boost in these two common clinical scenarios represents important areas of future study for the optimization of adjuvant breast radiation.

## Background

A considerable proportion of patients with early-stage breast cancer are treated with breast-conserving surgery (BCS) followed by whole breast radiation (WBI). In this group, an additional dose of radiation—a boost—can be delivered in order to reduce the risk of local recurrence (1–8). There is variation of boost dose, planning technique, radiation modality, and sequence, but in general, in addition to WBI, a few additional fractions of radiation are delivered to the lumpectomy site (including postoperative seroma and surgical clips) in addition to a margin, using various radiation modalities including photon or electron beams (9).

However, studies have shown that the higher radiation dose associated with the addition of a boost may lead to worse cosmetic outcomes (10–12). In a recent Cochrane review, adding a boost led to worse cosmesis when scored by a review panel (OR 1.41, 95% CI 1.07–1.85), but no difference in cosmetic outcomes when scored by a physician (OR 1.58, 0.93–2.69) (10). Immink et al. (11) assessed long-term cosmetic outcomes of 348 patients enrolled in the European Organization for Research and Treatment of Cancer (EORTC) boost vs. no boost trial. At 3 years, there was no significant difference between the patients that received a boost and those who did not; however, over longer-term follow-up it became clear that addition of a boost increased the degree of fibrosis (11). Another, larger analysis that included over 3,000 patients from this same trial found similar results (12). Specifically, they found that after a 10-year follow-up, the addition of a boost led to increased rates of moderate or severe fibrosis (12). In an older study that included just over 100 patients, addition of a boost was linked to other long-term side effects such as telangiectasis and depigmentation (13). Beyond cosmetic outcomes, use of a boost adds to the cost of radiation therapy. Lanni et al. (14) estimated that the cost of WBI was US$11,725 using opposed tangents and US$20,637 with 3D-CRT/IMRT. With the addition of a boost, this increased to $13,829 and $22,130, respectively (14).

Therefore, radiation boost should be reserved for patients whose potential benefit from additional radiation outweighs the risks and justifies the additional costs. Younger patients have consistently been shown to be at higher risk for local recurrence, with age acting as an independent risk factor (1–3, 5, 7, 15). Given this, they would be expected to benefit more from a boost, and this is what the EORTC boost vs. no boost trial demonstrated. In this study, following whole breast radiation (50 Gy/25 fractions), patients were randomized to receive a boost of 16 Gy to the tumor bed or no boost (1). In younger patients, the addition of a boost translated into a significantly higher absolute risk reduction in comparison to older patient groups (1). For patients ≤ 40 and 41–50 years of age, the absolute reduction in risk of local recurrence was 11.6 and 5.9%, as compared to 2.9 and 3.0% for patients 51–60 and >60 years of age, respectively (1). As far as side effect profile is concerned, rates of fibrosis were higher in the boost group for all age groups except for patients ≤ 40 (1). Hazard ratios for fibrosis by age group were 1.02 (99% CI 0.17–6.22, *p* = 0.98), 3.51 (1.16–10.55, *p* < 0.003), 3.15 (1.49–6.65, *p* < 0.001), and 2.55 (1.24–5.27, *p* < 0.001) for patients age ≤ 40, 41–50, 51–60, and > 60.

The benefit of boost in younger patients is appropriately reflected in the pattern-of-practice data, where age exerts a strong influence on the decision to add a boost (16–18), as well as in the guideline recommendations from collaborative groups and national agencies (Table 1). Within these guidelines, age is the most consistently cited factor, with most using a cut-off of 50 years. However, beyond age, other determinants of boost utilization such as tumor grade, presence of lymphovascular invasion (LVI), hormone receptor status, and presence of positive margins are not supported by high-level evidence, creating the potential for variation in recommendations and practice, as reflected in the available guidelines (Table 1).

**Table 1:** Summary of guidelines and expert recommendations on the indications for adding boost radiation.

**Organization**	**Recommendations**
ASTRO (American Society for Radiation Oncology)—(19)	Boost is recommended for: • ≤ 50 years old • 51–70 years old with high-grade tumor • Positive margins• Omitting boost is recommended for: • >70 years old with low or intermediate grade, hormone positive tumor that was excised with a widely negative margin (≥2 mm)• Boost and fractionation schedule: • Use of a boost should be independent of the whole breast fractionation scheme.
GEC-ESTRO Breast Cancer Working Group—(20)	Boost may be omitted for: • ≥50 years old with a ≤ 3-cm unicentric, unifocal tumor with no nodal involvement that was resected with a widely negative margin (≥2 mm), with no LVI or no EIC (extensive intraductal component) and not triple negative• Boost with dose escalation (above 16 Gy EQD2) is recommended for: • ≤ 40 years old with close margins, EIC or triple negative disease • Positive margins• Boost with or without dose escalation is recommended for: • ≤ 40 years old and do not meet criteria for boost with dose escalation • 40–50 years old • >50 years old with any of the following risk factors (close margins, tumor >3 cm, extensive intraductal component, LVI, node involvement, multicentric or multifocal disease, triple negative disease, or residual disease after neoadjuvant chemotherapy)
Consensus from 15th St. Gallen Expert Conference, ESMO (21)	Boost may be omitted for: • >60 years old with low-grade tumor and/or favorable tumor biology who will be receiving adjuvant endocrine therapy.
ESMO (European Society for Medical Oncology)—(22)	Boost is recommended for: • <50 years old • Grade 3 • Extensive DCIS • LVI • Focally positive margin
SSO-ASTRO Consensus Guideline (23)	Boost is not necessarily recommended for: • Close margins. More specifically, use of a boost should be based on “a priori estimation of IBTR [ipsilateral breast tumor recurrence] risk and should not be determined, in isolation, by the width of the surgical margin”
National comprehensive cancer	Boost is recommended for: • Patient at higher risk of recurrence
network (NCCN)—(24)	Boost with consideration of dose escalation is recommended for: • Microscopically focally positive margins, in the absence of EIC

This review will focus on two other important factors for boost decision-making, namely, close surgical resection margin status and fractionation schedule. We will review the available evidence as well as the patterns-of-practice data surrounding each.

## Margin Status, Close

Surgical margin is the width of non-cancerous tissue surrounding the tumor when resected, with a general concern that a narrow (close) but negative margin might correlate with increased risk of recurrence. The exact definition of close margins for breast cancer resected with BCS has been variable; however, the most commonly used definition for close margins in invasive breast cancer is <2 mm (25).

Management of patients with close margins in invasive breast cancer has undergone a recent shift in practice. Previously, re-excision was recommended for both close and positive margins; however, recent evidence has demonstrated that once there is no “tumor on ink,” increasing the margin width does not correlate with reduction in local recurrence, and therefore, consensus practice has moved to reserving re-excision for positive margins only (5, 25–27).

In light of this, guidelines by Society of Surgical Oncology—American Society for Radiation Oncology (SSO-ASTRO) recommended that the decision to deliver a boost be based on overall assessment of the risk of local recurrence and that the width of margins is not, in and of itself, an indication for a boost (23). To date, there is no new evidence since the SSO-ASTRO recommendations in 2014 that convincingly suggests that width of negative margins should dictate the decision to add a boost when treating invasive breast cancer.

Looking closer at the available literature, a meta-analysis by Houssami et al. (27) which investigated the effect of margin status and width on cancer outcomes found that after addressing differing rates of boost as a possible confounder, the width of the negative margin did not significantly affect local control (*p* = 0.86). This analysis included 33 separate studies reporting on a combined 32,363 patients (27). Comparable results were found in a recent analysis by Vrieling et al. of the EORTC boost vs. no boost trial (28). For inclusion in this trial patients were required to have a negative margin (i.e., no tumor on ink) as assessed by a local pathologist. However, 1,616 patients, or 30% of the study population, then underwent a central pathology review which re-assessed margin status (28, 29). In Vrieling et al. (28), margins confirmed as negative on pathology review were divided by width of the negative margin ( ≤ 5, 3–4, or ≤ 2mm). The rate of boost in each margin category was roughly similar, with a boost used in 52% (497/950), 49% (91/187), and 48% (146/306), for ≥5, 3–4, and ≤ 2-mm (28). Over a median follow-up of 18.2 years the rate of local relapse as first event was 10% (95/950), 11% (20/187), and 9% (29/306) for ≤ 5, 3–4, and ≤ 2 mm (28). These rates were not significantly different with a hazard ratio for local relapse by margin statuses of 1 (reference), 1.10 (95% CI 0.68–1.78), and 0.97 (0.64–1.47), for ≥5, 3–4, and ≤ 2 mm (28). Here, each group received a boost at similar frequencies and demonstrated similar rates of local recurrence over long-term follow-up, which supports the idea that the use of a boost is not a confounding factor in the excellent local control rates seen in close margin resections that do not undergo re-excision and that therefore close margins are not an indication for a boost.

The data from Vrieling et al. (28) also showed that the addition of a boost provided a similar reduction in risk of local recurrence for negative margin widths of ≥5 vs. 3–4 mm or ≤ 2 mm (*p* = 0.63). However, in an earlier analysis of the same data, Jones et al. (29) found that addition of a boost significantly reduced local recurrence in patients with negative margins >2 mm (HR 0.47, *p* = 0.0004), but not for patients with negative margins <2 mm or positive margins (*p* = 0.65). This study grouped <2-mm and positive margins together; however, there were relatively few patients with positive margins (29). Nevertheless, this may offer an explanation for the difference between Vrieling et al. (28) and Jones et al. (29). Additionally, Jones et al. (29) had a shorter median follow-up time of 10 years and it is possible that, with the shorter follow-up time, the effect of boost could not reach statistical significance for the <2-mm and positive margin group, which was roughly 4-fold smaller than the >2-mm group.

Next, turning to patterns-of-practice, the available data is somewhat limited. Ceilley et al. (30) surveyed 1,137 physicians and found that, among active physicians of ASTRO and ESTRO, there was a significantly increased likelihood to add a boost for close margins. Among the 702 American physicians surveyed, 85% gave a boost in patients with negative margins compared to 98% for patients with close margins (*p* < 0.001) (30). Data from European physicians was similar with 75% giving a boost in cases of negative margins vs. 94% for close margins (30). However, this survey data was collected in 2002, which is prior to the shift away from re-excision for close margins (30). Looking at more recent studies, Nguyen et al. (17) surveyed 388 radiation oncologists in Australia and New Zealand, receiving responses from 156 of them. They found significant division in opinion around close margins as an indication for a boost. 35.2% felt that a margin <2 mm was an absolute indication for a boost, 38.7% felt it was a relative indication, and 26.1% felt it was not an indication (17). Although limited, available data does suggest that significant variations in practice exist with regards to a boost for close margins.

## Fractionation Schedule

The most recent ASTRO consensus guidelines support the use of hypofractionated whole breast radiation (40 Gy/15 fractions or 42.5 Gy/16 fractions) for the vast majority of patients. Specifically, they support its use for any age group, in combination with any chemotherapy regimen, and for patients with any stage of disease, provided that they do not require coverage of regional lymph nodes (19). However, pattern-of-practice studies show an interesting trend toward far lower rates of boost utilization when using hypofractionation vs. conventional fractionation. Stokes et al. (16) analyzed patterns of practice for patients with early-stage breast cancer treated between 2004 and 2014 using the US National Cancer Database (NCDB), identifying a total of 423,500 patients. They found that those managed with hypofractionation received a boost significantly less frequently (OR 0.15, 95% CI 0.15–0.16, *p* < 0.001). Another analysis of the NCDB by Zhong et al. (18) which included 356,160 patients showed similar results, with a boost being given in 88.9% of the cases following conventional fractionation vs. 52.2% of the time after hypofractionation (*p* < 0.001).

Looking at the evidence for addition of boost with hypofractionation vs. conventional fractionation, there does not appear to be significant differences in clinical outcomes. Addressing long-term cosmetic outcomes first, the addition of a boost appears to lead to worse cosmetic results regardless of fractionation schedule (31, 32). De Santis et al. (32) compared toxicity outcomes following hypofractionated WBI with 42.4 Gy in 16 fractions with or without a boost and on univariant analysis boost was a significant predictor of late toxicity (*p* < 0.001). This is similar to the trend toward worse cosmetic outcomes with addition of a boost within conventional fractionation (10–12).

More importantly, cosmetic outcomes are similar when a boost is used in combination with hypofractionated vs. conventional WBI (33–39). In their 120-patient study comparing addition of a boost to conventional fractionation (50 Gy/25 fractions) vs. hypofractionation (42.5 Gy/16 fractions), De Felice et al. (34) found no difference in long-term breast fibrosis. Median follow-up in this study was 16 months (34). Similarly, in their 287-patient study, Shaitelman et al. (36) found no difference in any ≥ grade 2 or ≥ grade 3 toxicity, hyperpigmentation, skin induration, dermatitis, telangiectasia, fibrosis, or breast edema, at 6 months post-radiation with conventional WBI plus boost vs. hypofractionated WBI plus boost. Furthermore, a systematic review and meta-analysis by Valle et al. (39) found no significant difference (RR 0.95, 95% CI 0.81–1.12) in the rates of poor cosmetic outcomes between hypofractionation vs. conventional fractionation.

Next, with regards to cancer control, conventional and hypofractionation regiments have been shown to produce similar outcomes (33). Within conventional fractionation, the improved local control provided by a boost is well-documented (40). However, there is insufficient data specifically assessing the addition of a boost to hypofractionated WBI. From the UK START trial, a *post-hoc* analysis showed that in patients who received a boost, the rate of local–regional relapse was not significantly different between those treated with hypofractionated vs. conventional WBI (HR 0.99, 95% CI 0.76–1.29) (41). This suggests that the boost effect was similar in both dosing schedules. An older study by Romestaing et al. (8) using an alternative dosing schedule of 50 Gy in 20 fractions given over 5 weeks also showed decreased local recurrence with the addition of a boost. The dosing schedule used here is different from both conventional and hypofractionation schedules used today; however, it does support the assumption that a boost will provide improved local control regardless of the whole breast dosing schedule it is combined with.

Returning to the patterns-of-practice data, an important caveat to interpreting the results previously discussed is that patient characteristics may not have be similar between the groups treated with hypofractionated vs. conventional radiation. Using the same National Cancer Database (NCDB) as Stokes et al. (16) and Zhong et al. (18), Hasan et al. (42) found that hypofractionation was used more commonly in older patients (2.6% age <40 vs. 19.5% age >80; RR 8.40, 95% CI 5.01–14.09), those with node-negative disease (9.8% pN0 vs. 3.3% pN1; RR 0.38, 0.36–0.40), smaller tumors (9.5% ≤ 2.0 cm vs. 5.9% 2.1–5.0 cm; RR 0.78, 0.75–0.81), and lower-grade cancers [10.9% Grade 1 vs. 9.1% Grade 2 (RR 0.87, 0.85–0.90) vs. 5.8% Grade 3 (RR 0.79, 0.76–0.83)]. However, a survey of 2,150 randomly selected members of the American Society for Radiation Oncology suggests that the decision to forgo boost may still be made solely on the basis of WBI fractionation schedule. They reported that 94.4% of physicians used a boost after conventional fractionation in more than two thirds of their patients, compared to 14.4% after hypofractionation (43). Moreover, 69.7% indicated never using a boost after hypofractionation compared to 0% after conventional fractionation (43). This variation in the use of a boost based on fractionation schedule is in opposition to the most recent guideline by ASTRO which recommends that the decision to add a boost should be independent of the whole breast fractionation scheme (19).

## Discussion

Addition of a boost is an established technique for improving local control in higher-risk patients. However, improved local control can come at the cost of worse cosmetic outcomes (1, 4, 6, 7, 10–12). There is a lack of consensus between published guidelines on exactly which patients benefit from a boost, and largely, the decision is left to the discretion of individual physicians with or without the guidance of institutional policies and guidelines.

Here we have discussed the differences between consensus guidelines, patterns of practice, and current evidence surrounding use of a boost with regards to close margins and WBI fractionation. Due to the recent practice changes around re-excision for close margins, there is not good long-term data on the local recurrence of close margins with or without a boost. The overall consensus of guidelines indicate that close margins are not, by themselves, an absolute indication for a boost; however, in at least some of the recent guidelines, they do appear to be an important consideration in decision-making (19, 20, 23). As to how this is being implemented in day-to-day practice, this is unclear since our pattern-of-practice data is very limited. However, it is easy to imagine that there is a strong potential for practice variation.

In order to minimize these variations in guidance and practice, we will eventually need more long-term data assessing local recurrence with and without a boost for patients with close margins preferably from prospective studies, also incorporating our modern understanding of tumor biology, particularly as we move into a time when close margins are not routinely re-excised. In the meantime, studies to understand the practice pattern for boost utilization with close margins could offer insight into how these patients are being managed.

With the current state of evidence, however, it is perhaps most reasonable to follow the guidance in Moran et al. (23) and not to use close margins as a sole indication for a boost, but to base the decision on an overall assessment of the risk of local recurrence. This unfortunately is somewhat vague. Moreover, the current status of evidence leaves the possibility that close margins may exert a more significant effect on the gestalt impression of risk of recurrence than is truly warranted, especially since current data suggests that patients with close margins have excellent local control rates similar to those with wide negative margins, regardless of the use of a boost.

As for hypofractionation, although there is specific guidance indicating that the addition of a boost be independent from the whole breast fractionation schedule (19), it appears that patterns of practice may not entirely follow consensus guidelines. Instead, the data shows that a boost is used far less frequently in cases of hypofractionation, at least at some jurisdictions. The reason for lower utilization of a boost in hypofractionation could be from concern about inferior cosmesis. However, the current evidence shows similar toxicity profile and benefit for a boost with conventional vs. hypofractionated WBI. Therefore, the lower rates of boost utilization with hypofractionation represent an area of potential future research focus to support practice. Further studies specifically on the effect of adding a boost to hypofractionation will help elucidate this issue, but it will take years for relevant outcomes data to become available. In the meantime, it seems most reasonable to make decisions on addition of a boost independent from fractionation schedule.

## Author Contributions

HR provided supervision and guidance to SG, who was responsible for the literature review and drafting of this article.

## Conflict of Interest

The authors declare that the research was conducted in the absence of any commercial or financial relationships that could be construed as a potential conflict of interest.
